# Biorefinery Concept Employing *Bacillus coagulans*: LX-Lignin and L-(+)-Lactic Acid from Lignocellulose

**DOI:** 10.3390/microorganisms9091810

**Published:** 2021-08-25

**Authors:** Linda Schroedter, Friedrich Streffer, Katrin Streffer, Peter Unger, Joachim Venus

**Affiliations:** 1 Bioengineering Department, Leibniz Institute for Agricultural Engineering and Bioeconomy e. V. (ATB), 14469 Potsdam, Germany; punger@atb-potsdam.de; 2 LXP Group GmbH, 14513 Teltow, Germany; friedrich.streffer@lxp-group.com (F.S.); katrin.streffer@lxp-group.com (K.S.)

**Keywords:** *Bacillus coagulans*, biorefinery, corn stover, lactic acid, lignin, lignocellulose, rye straw

## Abstract

A new biorefinery concept is proposed that integrates the novel LX-Pretreatment with the fermentative production of L-(+)-lactic acid. Lignocellulose was chosen as a substrate that does not compete with the provision of food or feed. Furthermore, it contains lignin, a promising new chemical building material which is the largest renewable source for aromatic compounds. Two substrates were investigated: rye straw (RS) as a residue from agriculture, as well as the fibrous digestate of an anaerobic biogas plant operated with energy corn (DCS). Besides the prior production of biogas from energy corn, chemically exploitable LX-Lignin was produced from both sources, creating a product with a low carbohydrate and ash content (90.3% and 88.2% of acid insoluble lignin). Regarding the cellulose fraction of the biomass, enzymatic hydrolysis and fermentation experiments were conducted, comparing a separate (SHF), simultaneous (SSF) and prehydrolyzed simultaneous saccharification and fermentation (PSSF) approach. For this purpose, thermophilic *B. coagulans* 14-300 was utilized, reaching 38.0 g L^−1^ LA in 32 h SSF from pretreated RS and 18.3 g L^−1^ LA in 30 h PSSF from pretreated DCS with optical purities of 99%.

## 1. Introduction

Biorefineries are evolving, since industry and consumer demand for materials made from renewable resources is continually on the rise. Coupled with sufficient political incentives, the bioeconomy is expected to be thriving in the next few decades [1,2]. Similar to the vast expansion of the petrochemical product range, biorefineries are anticipated to diversify their production lines and establish sustainable platform technologies. To meet this objective, the need for stronger connections between feedstock, conversion technologies and end usage of the obtained products has been emphasized [1]. Whilst starch or sucrose-containing feedstocks (e.g., corn, sugar cane) are the main sources for industrial biotechnology at present, alternative feedstocks are increasingly coming into focus of research and innovative enterprises [3,4,5].

Lignocellulose is one example of such a novel substrate: It is the part of the plant that provides a structure as well as resilience against several natural stresses (e.g., mechanical, chemical or microbial stresses). To provide these abilities, lignocellulose comprises of the three chemical structures cellulose, hemicellulose and lignin, whose proportions and composition differ for each plant, as well as the plant’s part, age, cultivation conditions or time of harvest [6,7]. Cellulose essentially consists of crystalline fibers from glucose monomers and is encompassed by a heteropolymer hemicellulose network that contains varying pentose and hexose sugars (e.g., xylose, arabinose, galactose) [8]. Lignin is a very complex polymeric structure of three phenolic compounds that build covalent bonds with the hemicelluloses [9,10]. When separating the structures, sugars of the cellulose and hemicellulose can serve as substrates for fermentation processes, while lignin itself is expected to become a groundbreaking chemical building material. It is anticipated that various novel chemical routes and products can be established from chemically valuable lignin [9,10,11].

Due to its innate robustness, biomass fractionation is one of the major challenges when lignocellulose is processed [12,13]. While lignin utilization in industrial applications is currently often limited to heat and power production, recent studies on pretreatment strategies and biorefineries focused on complete biomass utilization and aimed to achieve the selective fractionation of lignin [14,15]. About a decade ago, cellulose solvent- and organic solvent-based lignocellulosic fractionation processes (COSLIF) emerged and were addressed as forms of “feedstock-independent biomass pretreatment” [16,17]. Recently, the LXP Group GmbH (LXP) optimized the involved precipitation process and reduced the cellulose related operating materials, thereby facilitating their recovery (LX-Pretreatment) [18]. Furthermore, a lignin recovery process was suggested which replaces the burning of the lignin and allows its extraction as a valuable side stream with a low carbohydrate and mineral content [19,20].

The options for biorefinery concepts from lignocellulose are vast, considering the wide range of biotechnological and chemical production routes that have been pointed out in several reviews [4,9,11]. Most importantly, concepts that aim to develop valuable products from lignin alongside the production of bioethanol have been proposed [21,22,23,24,25]. In this study, the cascade utilization of lignocellulosic biomass for the production of valuable lignin and L-(+)-lactic acid is presented, to our knowledge, for the first time. Considered to be one of the main chemical building blocks for establishing a sustainable bio-based economy [26], lactic acid (LA) has many applications ranging from the food, pharmaceutical and, more recently, to the polymer industry. Its stereoisomeric forms L-(+)- and D-(−)-LA are used for the production of polymer lactic acid (PLA), an alternative, non-fossil based compound for the production of plastic goods [27,28].

The study presented here was financed by the European Regional Development Fund, which is especially designed to support small or medium-sized enterprises (SMEs) and to connect research to innovations that promote a low-carbon economy. The scope of the project was the production of two valuable products from lignocellulosic residues: Lignin and optical pure L-(+)-LA as sustainable platform chemicals. Two different feedstocks from established processes were tested for this application: rye straw (RS; residue from agriculture) on the one hand and the digestate of energy corn silage (DCS; residue of an anaerobic biogas plant) on the other hand. Both substrates are grown regionally, which is logistically advantageous when planning a biorefinery in Germany. Within Europe, about 92% of the rye produced worldwide is cultivated. Germany is the leading producer with 3528 kt per year on an area of 627,000 hectares (projection for 2021) [29]. Regarding the cultivation of energy crops for biogas production, 9.7% of German agricultural land was used in 2020. The main share of 64% is energy corn with a cultivated area of 989,000 hectares [30]. The LXP Group GmbH focused on the production of LX-Lignin, conducting their innovative, patented LX-Pretreatment. The generated process residue, designated as LX-Cellulose, was handed to the research facility ATB for the biotechnological production of L-(+)-LA, employing the bacterial strain *Bacillus coagulans*.

The thermophilic nature of *B. coagulans* enables the testing of simultaneous enzymatic hydrolysis and fermentation approaches, since the optimum of the applied enzymes usually lies at elevated temperatures. In addition, an increased temperature lessens the risk of contamination by mesophilic microorganisms, thereby allowing processes to be conducted with a reduced necessity of sterilization [31]. Furthermore, *B. coagulans* is able to consume both hexoses and pentoses as well as tolerate inhibitor compounds derived from pretreated lignocellulose [32,33], while nutrition requirements remain simple [34,35]. Considering all this, *B. coagulans* qualifies as an excellent candidate for the fermentation of lignocellulosic substrates. For this study, three different fermentation approaches were compared to find the optimal experimental set-up for further investigations: Separate hydrolysis and fermentation (SHF), simultaneous saccharification and fermentation (SSF) as well as prehydrolyzed simultaneous saccharification and fermentation (PSSF).

The exploitation of the lignocellulosic feedstock is proposed in a biorefinery concept: A value-adding cascade from renewable feedstock was established, achieving up to three valuable products from one source. Additional to the rye harvested for food and feed and the biogas produced from energy corn, process residues could successfully transformed into LX-Lignin and L-(+)-LA by the novel LX-Process combined with microbial fermentation of *B. coagulans*.

## 2. Materials and Methods

### 2.1. Raw Material and Processing

Rye straw was kindly provided by a local farmer (Liebenwalde, Germany). The straw was chopped to lengths of 3–8 cm, similar to conditions used in animal bedding, and was stored at room temperature prior to usage for the LX-Pretreatment. Fibrous digestate of an anaerobic biogas plant was kindly provided by the operator (Ketzin, Germany). The plant was operated at elevated mesophilic temperatures, using energy corn silage as its main feedstock. The gained biogas was utilized in a combined heat and power unit (CHP), as well as upgraded to a natural gas quality and fed to the German energy grit. The corn silage digestate was used after air drying without further processing for the LX-Pretreatment. Provided digestate was stored at room temperature.

Alongside the project, the LX-Pretreatment underwent improvement and LX-Cellulose from both substrates was provided in several batches. Enzymatic hydrolysis and fermentation experiments presented in this publication were done from single batches of material, thereby ensuring comparability of the results.

### 2.2. LX-Pretreatment

RS and DCS underwent the LX-Process at approximately 80% dry matter content, gaining chemically exploitable LX-Lignin as well as solid residues with enhanced cellulose content designated as LX-Cellulose. The LX-Process has been described in detail in its patent [18]. Basically, the process comprises of the dissolution of the lignocellulosic biomass in 75–80% phosphoric acid at 50–75 °C and ambient pressure at a ratio of approximately 1:3 (*w/w*) for 15–45 min. After completion of the biomass dissolution, carbohydrates are precipitated. Various precipitants like acetone or ethanol have been described in the literature [36,37]. However, the LX-Process applies an agent of lower polarity, reducing its retention in the precipitated amorphous carbohydrates [18]. As a result, both the recovery of the phosphoric acid and the precipitant are enhanced and facilitated. Subsequent to the precipitation, a liquid/solid separation is carried out. The liquid containing the LX-Lignin is then conveyed to a distillation step: Here, LX-Lignin is obtained in a solid form while the precipitant can be recovered and recycled. The carbohydrate solids conversely pass through several counter current washing steps with water, leading to the valuable side stream of the process: LX-Cellulose. By collecting and purifying streams from the distillation and the washing step, the phosphoric acid and water are recycled. LX-Lignin has been tested for the production of phenolic resins as well as vanillin, leading to promising results. LX-Cellulose was provided by LXP to the ATB and stored at −20 °C prior to further usage.

### 2.3. Enzymatic Hydrolysis of LX-Cellulose

For a sufficient enzymatic hydrolysis of LX-Cellulose, preliminary shaking flask experiments were carried out, employing the enzymatic solution Cellic^®^ CTec2 (CCT2, 250 FPU mL^−1^; Novozymes, Copenhagen, Denmark). LX-Cellulose total dry solids (TS; 6.8, 9.0 and 11.2 TS%) and enzyme solution concentrations (0.2, 0.3 and 0.4 mL g^−1^ cellulose) were varied in these experiments. Enzymatic hydrolysis tests were carried out in a shaking incubator for 24 h at 50 °C and 150 rpm, using 0.3 L Erlenmeyer flasks with aluminum caps. Prior to the enzyme solution addition, the pH-value was adjusted to pH 5 with 4% NaOH. Based on the results of the shaking flask trials, enzymatic hydrolysis of LX-Cellulose was conducted at a larger scale, using a 2 L lab scale fermenter unit equipped with a 6 blade Rushton turbine. Lab scale enzymatic hydrolysis was carried out in duplicates for 24 h, with a working volume of 1 L at 50 °C, 400 rpm and pH 5 (pH adjustment with 20% NaOH). For subsequent fermentation in SHF, the enzymes were inactivated by heating the obtained hydrolysate up to 85 °C, with this temperature maintained for 10 min. The inactivation step, including the heating and cooling down of the hydrolysate, took roughly 2 h. For practicability, the hydrolysate was kept at 7 °C overnight and used for fermentation on the next morning.

### 2.4. Bacterial Strain and Preculture Conditions

After a strain screening in the filtrate of DCS-LX hydrolysate, *Bacillus coagulans* DSM ID 14-300 (abbr. 14-300) was selected as the candidate for the fermentation experiments (published elsewhere [38]). Precultures for fermentation experiments were grown in 180 mL MRS (Merck Millipore, Darmstadt, Germany), added with 2 g EVERZIT^®^ Dol (0.5–2.5 mm sized CaCO_3_ · MgCO_3_; Evers, Hopsten, Germany) and kept in a shaking incubator for 15 h at 40 °C and 100 rpm.

### 2.5. Batch Fermentation

Batch fermentations were carried out in three different modes. First, a separate hydrolysis and fermentation (SHF) was conducted. Secondly, simultaneous saccharification and fermentation (SSF) was tested, adding a preculture and an enzyme solution at the same time. In the third experimental set-up, the enzymatic hydrolysis was initiated 12 h prior to the preculture addition (PSSF; this is also sometimes referred to as combined hydrolysis and fermentation (CHF) in the literature). Experiments were conducted with 1 L working volume at 400 rpm in duplicates, employing 2 L lab scale reactors (glass fermenter, 1 × 6 blade Rushton turbine). The inoculation volume for all the experiments was 6% (*v/v*), 10 g L^−1^ of yeast extract was added as a nutrient supply and 20% NaOH was added for automatic pH-regulation. For SHF, the optimal fermentation parameters of *B. coagulans* 14-300 were chosen (52 °C and pH 6). Regarding the SSF and PSSF mode, optimal conditions with respect to the enzyme solution were applied, running the hydrolysis and fermentation simultaneously at 50 °C and pH 5. Experiments with DCS-LX were carried out using 11.2 TS% and an enzyme concentration of 0.3 mL g^−1^ cellulose (or 27 FPU g^−1^ dry matter). Batches utilizing RS-LX were conducted with 10 TS% and 0.4 mL g^−1^ cellulose (or 67 FPU g^−1^ dry matter).

### 2.6. Analytics

Products of the LX-Pretreatment were analyzed according to protocols published by the National Renewable Energy Laboratory (NREL) [39,40,41]. Batches of RS-LX and DCS-LX destined for enzymatic hydrolysis and fermentation experiments were analyzed for neutral detergent fiber (NDF), acid detergent fiber (ADF) and acid detergent lignin (ADL) using the fiber analyzer ANKOMA^2000^ (Ankom Technology, Macedon, NY, USA). High performance liquid chromatography (HPLC; DIONEX, CA, USA) was conducted for the analysis of acid (LA, acetic acid) and sugar concentrations (glucose, xylose and arabinose) using a Eurokat H column (300 mm × 8 mm × 10 µm; Knauer, Berlin, Germany). Samples were diluted in water and filtrated into HPLC vials using a filter pore size of 0.20 µm. Injections of 10 µL were run within a mobile phase of 5 mM H_2_SO_4_ at a flow rate of 0.8 mL min^−1^ and detected via refractive index detector RI-71 (SHODEX, Tokyo, Japan). For the measurement of the LA optical purity, fermentation samples were diluted in the agent of the mobile phase, 2 mM CuSO_4_. Here, a Chiralpak^®^MA(+) column (50 mm × 4.6 mm × 3 µm; DAICEL, Osaka, Japan) and an ultraviolet detector were employed for the analysis via HPLC.

### 2.7. Calculations

From fiber analysis, the cellulose and hemicellulose fractions were obtained (NDF–ADF = hemicellulose, ADF–ADL = cellulose). The theoretical quantity of monosaccharides was calculated by using conversion factors from the literature (glucan/0.9 = glucose, xylan/0.88 = xylose) [8]. The enzymatic hydrolysis yield was calculated by dividing the total released monosaccharides by the theoretical quantity of monosaccharides present in the LX-pretreated substrate in g g^−1^. The fermentation yield was obtained by dividing the total produced LA by the available monosaccharides in the fermentation broth in g g^−1^. Thus, the dilution effect caused by adding the neutralizing agent as well as losses due to sample withdrawal were accounted for. Conversion efficiency was defined as the total produced LA divided by the theoretical quantity of monosaccharides present in the LX-pretreated substrate in g g^−1^. Average and maximum productivity of the fermentation were obtained by dividing the measured LA concentration by the fermentation time in g L^−1^ h^−1^.

## 3. Results

### 3.1. Analytics of Raw and LX-Pretreated Feedstocks

**Figure 1:**
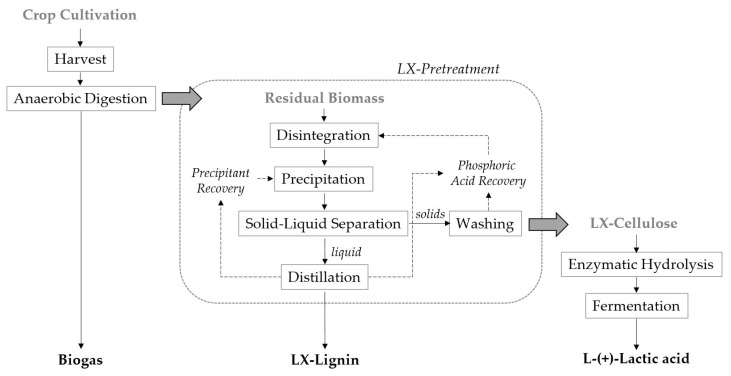
Schematic biorefinery process with an integrated LX-Process (LXP Group GmbH, Teltow, Germany); by following a value-adding cascade approach, several products can be gained from one renewable source.

Two substrates: RS and DCS, were pretreated with the patented LX-Process and the resulting side streams were tested in fermentation experiments. Figure 1 schematically displays the biorefinery process proposed by the joint project of the SME LXP and the research facility ATB. First, residual fibrous biomass from the anaerobic digestion of a biogas plant (or from rye harvest) was subjected to the LX-Pretreatment (see Section 2.2). The resulting LX-Cellulose was subjected to enzymatic hydrolysis to release fermentable carbohydrates out of the remaining, chemically opened structure of the biomass. The hydrolyzed slurry was then utilized for semi-sterile fermentation, employing the thermophilic microorganism *B. coagulans* 14-300. Following this concept, three valuable products, namely biogas, LX-Lignin and L-(+)-LA were obtained from energy corn and the value-adding cascade use of a renewable resource was realized.

Alongside the project, the LX-Pretreatment was optimized. From final process batches, the percentages of structural carbohydrates and lignin before and after the LX-Pretreatment were determined following the NREL procedures [39,40,41]. In Table 1, gathered data of the raw material as well as of the products LX-Lignin and LX-Cellulose are shown. Fractions of 90.3% and 88.2% of acid insoluble lignin were obtained from RS and DCS. Mineral and carbohydrate content of the LX-Lignin was favorably low, which indicates that it is a promising new starting material for the chemical industry. Pulp yields were not recorded for these experiments. However, comparable trials produced yields in the range of 60–80% of feedstock dry matter (data not shown). Usually, over 95% of the cellulose fraction is preserved during the LX-Process.

While 38.0% of cellulose could be determined in the RS feedstock, 60.1% glucan was present in the corresponding LX-Cellulose from RS. Therefore, the LX-Cellulose side stream demonstrated an enrichment of the glucan portion. For DCS, this proportion increased from 29.3% to 38.4%, respectively. Ash, hemicellulose, acid soluble lignin and acetic acid content decreased compared to the raw feedstocks. The reduced hemicellulose content of LX-Cellulose was mainly due to hydrolysis by the phosphoric acid. The hydrolysis could be influenced by changing process parameters such as the temperature: The higher the applied temperature, the lower the resulting hemicellulose content of the LX-Cellulose (data not shown).

This increase of the glucan portion is advantageous, considering its application for microbial fermentation. One of the drawbacks of lignocellulose fermentation is the limited ability of LA-producing microorganisms to consume pentose sugars. Many organisms that produce LA do not metabolize pentoses, or consume them inefficiently, being subject to carbon catabolite repression (CCR) [31]. Since many microorganisms preferentially take up glucose, an elevated glucan content in the substrate compared to the raw material is therefore of high value for the biotechnological production of LA. Another advantage of the LX-Pretreatment involves its moderate process conditions. Other processes for the disruption of lignocellulosic material, e.g., steam explosion, are conducted at elevated temperatures and/or pressures [7,42]. As a result, inhibitor compounds are generated, like furfural or hydroxymethylfurfural (HMF) that can hinder microbial growth [43]. The LX-Pretreatment was carried out at temperatures below 80 °C and under an atmospheric pressure. Furthermore, several washing steps of the LX-Cellulose were integrated to recover the phosphoric acid. Afterwards, only a few inhibitor compounds could be detected in the resulting LX-Cellulose. Early substrate batches in the project showed furfural and HMF concentrations of <10 mg L^−1^, combined [38]. Since the method was gradually improved alongside the project, no significant concentrations of inhibitor compounds were detected in later stage samples. Furthermore, up until now, no accumulation of HMF or furfural was observed in the operation of the technical center or demo plant of the LX-Process.

### 3.2. Enzymatic Hydrolysis of LX-Cellulose

To find a sufficient regime for the enzymatic hydrolysis of LX-Cellulose, preliminary shaking flask experiments were carried out varying the dry matter and the applied enzyme concentration. The aim was to find an experimental set-up resulting in high sugar concentrations while at the same time reducing the input of an enzyme solution and additional water. The dry matter content was limited to about 10–12 TS% to guarantee good continuous mixing. Considering the reduction of enzyme and water input will ultimately contribute to the economic viability of the whole process.

**Figure 2:**
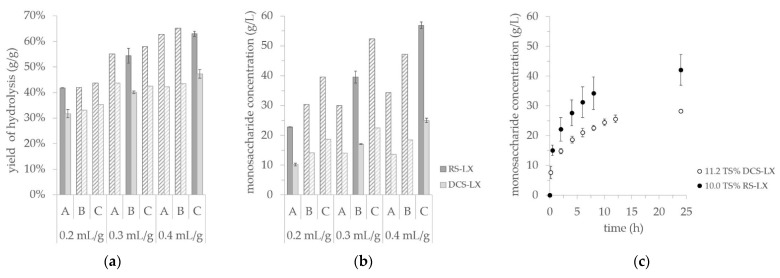
Enzymatic hydrolysis: (**a**) yield of hydrolysis and (**b**) monosaccharide concentration of shaking flask experiments with results from RS-LX and DCS-LX at 50 °C and pH 5 after 24 h duration: (A, B, C = 6.8, 9.0, 11.2 TS% load, enzyme concentration = 0.2, 0.3 and 0.4 mL CCT2 g^−1^ cellulose; crosshatching denotes experiments that were run without duplicates); (**c**) monosaccharide concentration of scale-up experiments in 2 L-benchtop reactors at 50 °C and pH 5 (0.3 mL CCT2 g^−1^ cellulose was added to the substrate DCS-LX and 0.4 mL g^−1^ to RS-LX, respectively).

Figure 2a,b displays the results of the optimization tests. Here, the hydrolysis yield and monosaccharide concentration after 24 h of enzymatic treatment are shown. Only certain settings were tested in duplicates for these preliminary trials. However, considering the low standard deviation of the duplicate trials, the entirety of the shaking flask results allowed us to conclude on settings for the subsequent fermentation trials.

It is noticeable from Figure 2a,b that an enzymatic hydrolysis of RS-LX produces higher values in comparison to experiments with DCS-LX. The highest monosaccharide concentration gained by the enzymatic hydrolysis of RS-LX was 56.9 g L^−1^ by employing 0.4 mL CCT2 g^−1^ cellulose at 11.2 TS%. The similar treatment of DCS-LX yielded 25.0 g L^−1^, respectively. Also, the hydrolysis yield for the tests with DCS-LX turned out to be lower, reaching 47.3% after 24 h of the theoretical sugars compared to 63.0% when RS-LX was hydrolyzed.

For one thing, the reduced sugar concentration could be explained by the lower initial cellulose content of DCS LX-C. However, the disparity of the two substrates regarding their hydrolysis yield might originate from the residual lignin measured in the samples, which was slightly increased for DCS-LX (compare Table 1). The negative effect of lignin during enzymatic hydrolysis can result from lignin binding to the enzymes or cellulose, which then blocks the enzymes from reaching the cellulose bound sugars [14].

Based on the results of the shaking flask, a specific enzymatic hydrolysis setting was chosen for each substrate. This configuration was conducted in 1 L working volume and was kept unaltered for the following separate and simultaneous fermentation approaches (SHF, SSF and PSSF). Figure 2c shows the results of these enzymatic hydrolysis experiments. For the material DCS-LX, the best setting of the shaking flask experiments was the application of 0.4 mL CCT2 g^−1^ cellulose at 11.2 TS%. Reduction of the enzyme solution to 0.3 mL CCT2 g^−1^ cellulose led to slightly reduced values of 22.5 g L^−1^ sugars and a yield of 42.4%. Considering the high costs that enzyme application adds to a process [3], the lower enzyme concentration was favored, accepting the decreased values of sugar concentration and hydrolysis yield. Conducting these experimental settings in 1 L working volume led to an average monosaccharide concentration of 28.3 g L^−1^ and 45.9% hydrolysis yield. These results were slightly higher than the values obtained by the shaking flask experiments.

Concerning substrate RS-LX, a load of 11.2 TS% again led to the highest monosaccharide concentration in the shaking flask experiments. However, regarding the hydrolysis yield, experiments for all the total solids percentages tested resulted in 63–65% at an enzyme charge of 0.4 mL CCT2 g^−1^ cellulose. The RS-LX substrate batch sufficed for 1 L working volume experiments of SHF, SSF and PSSF in duplicates if the total solid load was reduced to 10.0 TS%. Since it was assumed that the hydrolysis yield would still be sufficient, the reduction of TS load was favored over the option to reduce the working volume.

After performing an enzymatic hydrolysis of RS-LX in 1 L, an average monosaccharide titer of 42.1 g L^−1^ and hydrolysis yield of 51.0% was achieved. This hydrolysis yield result did not correlate with the assumption drawn from the shaking flask experiments. For one thing, scaling-up generally introduces changes to an experiment (e.g., altered mixing properties) and therefore can cause altered results. However, here, the heterogeneity of the RS-LX material should be taken into account instead. In Figure 2c, a distinct higher statistical deviation can be noted for the experiment utilizing RS-LX in comparison to the work with DCS-LX. Though the received substrate batch was thoroughly mixed, heterogeneity within the substrate sample could not be excluded at this stage of the process development. Thus, the presented enzymatic hydrolysis experiments should be understood as a preliminary investigation. With a similar pretreatment process and substrate compositions, hydrolysis yields of above an 80% glucan conversion can be achieved [44]. Therefore, an optimization of the enzymatic hydrolysis step should be repeated when the finalized LX-Cellulose pilot scale product is at hand. Furthermore, the chosen enzyme solution input for the work with RS-LX is rather high (67 FPU g^−1^ TS) and might not be economically feasible to use. Hence, for the enzymatic hydrolysis optimization of the final LX-Cellulose product, the economic viability of the whole process should be analyzed.

### 3.3. Lab-Scale Fermentation Experiments

**Figure 3:**
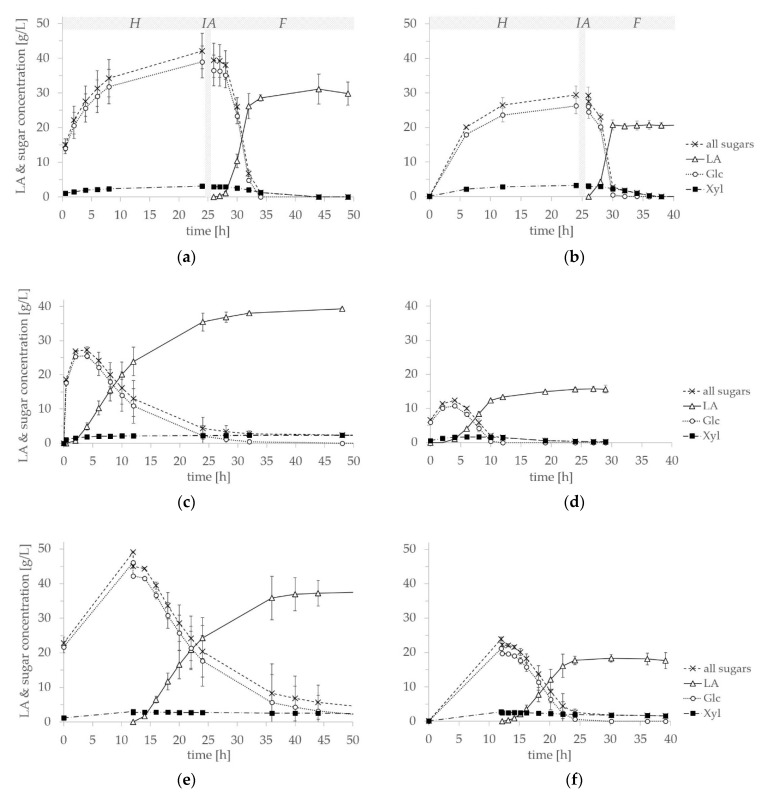
Separate and simultaneous fermentation experiments (*B. coagulans* 14-300, 1 L working volume, 10 g L^−1^ YE): (**a**) SHF of RS-LX and (**b**) SHF of DCS-LX, enzymatic hydrolysis (*H*) at 50 °C and pH 5, enzyme inactivation (*IA*) at 85 °C, fermentation (*F*) at 52 °C and pH 6; (**c**) SSF of RS-LX and (**d**) SSF of DCS-LX at 50 °C and pH 5; (**e**) PSSF of RS-LX and (**f**) PSSF of DCS-LX at 50 °C and pH 5.

Both the RS- and DCS-LX substrates were tested in fermentation experiments, employing thermophilic *B. coagulans* 14-300 for the production of L-LA. In Figure 3a,b results of the SHF experiments for RS- and DCS-LX are shown. For RS-LX, the enzymatic hydrolysis scale-up was directly utilized for fermentation. Here, the initial average concentrations of glucose and xylose were 39.0 and 3.1 g L^−1^, displaying shares of 92.5% and 7.5%. After inactivating the enzymes for 10 min at 85 °C, *B. coagulans* 14-300 preculture was added to the substrate. After 18 h of fermentation, a LA concentration of 31.1 g L^−1^ was measured, corresponding to an average productivity of 1.73 gL^−1^ h^−1^ and a fermentation yield of 84%. The fermentation was complete and all the monosaccharides were entirely consumed, while the overall process took 44 h. Performing the SHF process with DCS-LX led to an average monosaccharide concentration of 29.5 g L^−1^ after 24 h of enzymatic hydrolysis (see Figure 3b). The glucose concentration was 26.3 g L^−1^, accounting for 89.2% of monosaccharides. For xylose a concentration of 3.2 g L^−1^ was measured, representing 10.8% of the released sugars, respectively. Again, an inactivation step was performed and 20.7 g L^−1^ LA were produced within 4 h of fermentation. At this point of fermentation, 2.3 g L^−1^ of xylose was still present in the medium. By continuing the fermentation, a complete consumption of the xylose could be achieved, but the lactic acid concentration was not enhanced, leading to a productivity of 1.72 g L^−1^ h^−1^ after 12 h of fermentation. A maximum LA concentration of 21.1 g L^−1^ LA was measured after 24 h of fermentation and 50 h of process time, resulting in a fermentation yield of 84%. 

For the fermentation of glucose, the facultative anaerobic *B. coagulans* utilizes the Embden-Meyerhof-Parnas (EMP) pathway, which converts the hexose efficiently into LA. Unlike several lactic acid bacteria (LAB) that use the hetero-fermentative phosphoketolase pathway (PKP) for the consumption of xylose, *B. coagulans* metabolizes xylose via homo-fermentation. In this pentose phosphate pathway (PPP), LA is formed as the main product with negligible formation of by-products, while the PKP results in equal molar amounts of LA and acetic acid [45]. *B. coagulans* prefers the consumption of glucose over xylose, leading to a higher productivity during glucose metabolization. As soon as the glucose is fully consumed, the lower rate of xylose metabolization leads to a second fermentation phase with decreased productivity.

In a second experimental set-up, the simultaneous approach was tested. Here, the inactivation step was omitted and both enzymatic hydrolysis and fermentation were started at the same time. The results of SSF experiments are shown in Figure 3c,d. Utilizing RS-LX for SSF, the maximum LA concentration of the SHF process was surpassed in less than 24 h of process duration, reaching 35.5 g L^−1^ (1.48 g L^−1^ h^−1^ productivity). An average maximum concentration of 39.3 g L^−1^ LA could be achieved within 48 h, reaching 0.82 g L^−1^ h^−1^ productivity. This corresponds to an increase of 26.4% of maximum LA titer in comparison to the SHF approach. However, the xylose concentration in the substrate did not decrease over the course of fermentation, leading to a remaining residual xylose concentration of 2.3 g L^−1^ in the medium. For SSF with substrate DCS-LX, a different course of fermentation could be noted. Here, an elongated lag-phase occurred and the maximum LA titer of the SHF experiments could not be reached, let alone exceeded. The best result of 15.7 g L^−1^ LA concentration was measured after 27 h of SSF, resulting in a productivity of 0.58 g L^−1^ h^−1^. Here, no xylose could be detected at the end of the fermentation.

The observation of decreased LA production during SSF of DCS-LX can be explained by the presence of lytic polysaccharide monooxygenases (LPMOs) in the formulation of Cellic^®^ CTec2 [46]. LPMOs support crystalline cellulose degradation by oxidative cleaving of β−1,4 glycosidic bonds. While being advantageous for the saccharification of the substrate, a competition for dissolved oxygen between LPMOs and microorganisms can occur during fermentation. Employing *B. coagulans* for SSF of pretreated birchwood reduced the functionality of LPMOs and negatively affected saccharification [46]. Concerning the results of the work with RS-LX, no suppression of LPMO activity nor a negative impact on saccharification during SSF was indicated. Here, the SSF experiment produced higher lactic acid titers compared to SHF. It is possible that the competition for dissolved oxygen between LPMOs and *B. coagulans* at the beginning of the fermentation was shifted by providing a higher enzyme load. However, this theory should be proven by experiments that include the measurement of LPMO activity.

Additionally to the reduced LA production, the elongated lag-phase indicated that the released sugars might not be accessible for the organisms at the beginning of SSF with DCS-LX. Therefore, a third approach was investigated, which allowed a 12 h prehydrolysis of the substrate material. The goal in the PSSF approach was to provide sufficient time for the enzymatic hydrolysis whilst still saving time and experimental effort in comparison to the SHF process. In regard to LPMOs, a prehydrolysis was expected to postpone the loss of functionality and allow a higher yield of saccharification compared to the SSF process [46]. According to the enzymatic hydrolysis experiments, a time point of 12 h was chosen. Here, around 90% of the total released sugars had already gone into the solution (compare Figure 2c). Thereupon, the PSSF experiment was conducted, adding the preculture after 12 h of prehydrolysis to the substrate slurry.

The results of the PSSF processes are displayed in Figure 3e,f. In comparison to SSF, elevated sugar concentrations could be measured prior to the addition of the preculture. While 18.7 and 6.6 g L^−1^ monosaccharides were present at the start of RS- and DCS-LX SSF, concentrations of 45.0 and 22.2 g L^−1^ sugars were available after 12 h prehydrolysis in PSSF, respectively. Utilizing RS-LX for PSSF, a maximum concentration of 37.9 g L^−1^ LA was measured after 48 h of fermentation with a productivity of 0.79 g L^−1^ h^−1^ and an overall process time of 60 h. Similarly to the SSF process, the xylose concentration remained almost unchanged, resulting in 2.3 g L^−1^ residual sugars. For DCS-LX, a maximum LA concentration of 18.3 g L^−1^ was measured after 18 h of fermentation, resulting in a productivity of 1.02 g L^−1^ h^−1^. To achieve this, an overall process time of 30 h was needed. Again, parts of the xylose stayed unconsumed, leading to 1.8 g L^−1^ residual xylose at the time point of maximum LA concentration. Complete utilization of both hexose and pentose sugars could mainly be observed for the SHF experiments, which were conducted during the fermentation phase at pH 6. SSF of RS-LX and PSSF of both materials maintained residual xylose. In contrast to SHF, SSF and PSSF were conducted at pH 5 to provide the optimum pH-regime for the enzyme during the whole process. It is possible that at a low pH, *B. coagulans* 14-300’s ability to metabolize xylose is affected.

**Figure 4:**
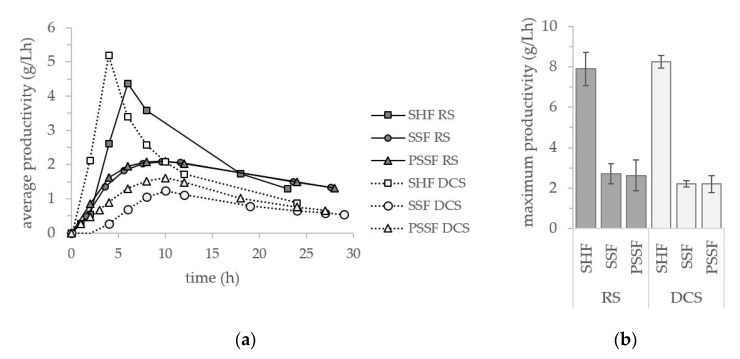
Productivity of *B. coagulans* 14-300 in SHF (52 °C and pH 6), SSF and PSSF (50 °C and pH 5) fermentation of RS- and DCS-LX substrate: (**a**) average productivity, (**b**) maximum productivity.

For the comparison of all three fermentation performances, average and maximum productivities of *B. coagulans* 14-300 are displayed in Figure 4a,b. High average productivities of 5.18 and 4.37 g L^−1^ h^−1^ were achieved in SHF of DCS- and RS-LX until glucose was fully consumed. The remaining xylose was metabolized at a lower rate and therefore the average productivity of the SHF process decreased to 1.73 g L^−1^ h^−1^ for the consumption of the entire monosaccharides. In SSF and PSSF, the average productivities were reduced regarding both substrates. Compared to SSF, conducting PSSF of RS-LX had no impact on the average productivity of the process. Meanwhile for the work with substrate DCS-LX, the average productivity could be enhanced choosing PSSF over SSF. Regarding the maximum productivities, the highest values of 7.91 and 8.25 g L^−1^ h^−1^ were reached in SHF with RS- and DCS-LX, respectively (see Figure 4b). In SSF processes, the maximum productivity was significantly reduced, reaching an average 2.70 g L^−1^ h^−1^ for RS-LX and 2.20 g L^−1^ h^−1^ for DCS-LX. Elevating the time of prehydrolysis in PSSF, neither had an impact on the maximum productivity of *B. coagulans* 14.300 for the fermentation of RS-LX or DCS-LX.

**Figure 5:**
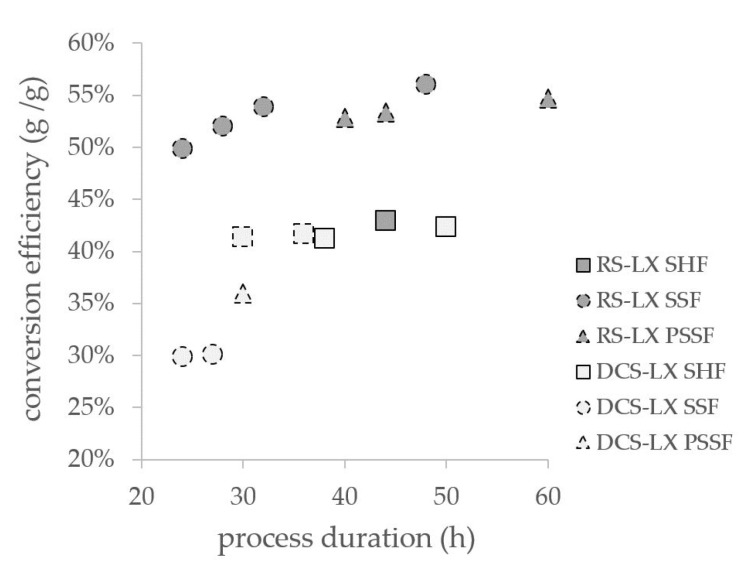
Process performance comparison for separate (SHF, 52 °C and pH 6) and simultaneous (SSF, PSSF, 50 °C and pH 5) fermentation approaches employing *B. coagulans* 14-300; dashed frames denote fermentation with incomplete sugar consumption (residual xylose).

In Figure 5, all conducted processes are compared by displaying the conversion efficiency against the overall process time needed. For substrate DCS-LX, the maximum average LA titer of 21.1 g L^−1^ was gained via SHF in a 50 h process time and corresponded to a conversion efficiency of 42.3%. All sugars were consumed at this point of time. SSF results indicated a reduced overall process time, but the conversion efficiency only reached 30.1% and a residual xylose concentrations of 0.3 g L^−1^ remained in the medium. In PSSF, 36.0% conversion efficiency could be achieved in 30 h of process duration. In comparison to the separate process at this time point, a slightly lower xylose concentration of 1.7 g L^−1^ was present in the medium instead of 2.3 g L^−1^ in SHF.

Utilizing substrate DCS-LX, the SHF process provided the highest LA titers. However, the simplification of the process through simultaneous fermentation strategies regarding effort and energy input should be taken into account when an overall biorefinery process concept is planned. A reduction in conversion efficiency might be tolerable when other process properties turn out to be economically advantageous.

For substrate RS-LX, a different outcome of the fermentation experiments could be determined. Here, the SSF approach brought several advantages, which could not be exceeded by performing a prehydrolysis of 12 h. Over 97% of the maximally produced LA was generated in 32 h of the SSF process with a productivity of 1.20 g L^−1^ h^−1^, corresponding to a conversion efficiency of 53.4%. Adding enzyme solution and preculture simultaneously saved time and experimental effort compared to SHF. However, the amount of applied enzyme dosage was comparably high and might not be economically feasible to use. Hence, though a good result could be achieved with RS-LX, the enzyme load should be further optimized in future experiments.

## 4. Discussion

In this study, a biorefinery concept is proposed, which aims at a complete lignocellulose biomass utilization. The integrated LX-Pretreatment allowed the selective fractionation of lignin, while also producing the LX-Cellulose side stream, which was tested for fermentation for the first time in this publication. Tolerance of a low pH-regime as well as the thermophilic nature of *B. coagulans* allowed the testing of simultaneous fermentation and saccharification approaches, working at the optimum process conditions of the employed enzyme solution (50 °C and pH 5). At first, experiments for the enzymatic hydrolysis of LX-Cellulose were carried out. For utilizing DCS-LX, 11.2 TS% with the addition of 0.3 mL CCT2 g^−1^ cellulose were chosen, as well as 10 TS% and 0.4 mL g^−1^ cellulose for the work with RS-LX. Subsequently, SHF, SSF and PSSF processes were conducted and compared for the two lignocellulosic substrates. Depending on the substrate, these tests led to different results: DCS-LX showed reduced values for LA titer and productivity in SSF compared to the separate process. However, allowing a prehydrolysis of 12 h before adding the preculture improved the results of the SSF in terms of the LA concentration, reaching 18.3 g L^−1^ in 30 h of overall process time with an optical purity of >99.9%. Here, residual xylose of 1.7 g L^−1^ remained in the medium. Full consumption of the monosaccharides released from DCS-LX was noted in SHF, reaching 20.6 g L^−1^ LA in 38 h overall process time with 99.5% optical purity. 

For substrate RS-LX, the best results in terms of LA concentration and overall process duration were indeed achieved by conducting SSF. With this fermentation approach, 38.0 g L^−1^ L-LA were produced in 32 h and a maximum titer of 39.3 g L^−1^ L-LA was measured after 48 h of the process (99% optical purity). Again, residual xylose remained in the medium, resulting in 2.3 g L^−1^ sugars at the maximum LA titer. Complete sugar consumption was only achieved by performing SHF, reaching 31.1 g L^−1^ LA in 44 h of overall process time.

Comparing the obtained results with recent publications, it primarily must be noted that titers of over 100 g L^−1^ LA were aimed for [47]. However, this is often only achieved when time- or energy-consuming strategies are used. For example, Wei et al. (2018) achieved 130.3 g L^−1^ L-LA when fermenting pretreated corn stover with the genetically modified *Pediococcus acidilactici* ZY271 after 6 h of prehydrolysis and 72 h of fermentation. Although the fermentation is rapid and it results in this high titer, the procedure is time consuming, since the process is preceded by three days of biodetoxification. Hereby, inhibitor compounds are reduced that would otherwise hinder the growth of the organism [48,49]. Another example for a high-titer fermentation from pretreated corn stover is the work from Ma et al. (2016). Within 33 h of fermentation, 98.3 g L^−1^ L-LA could be achieved by employing *B. coagulans* NBRC 12714. However, this high titer could only be obtained by separating the hydrolysate in advance by means of membrane filtration and concentrating it subsequently by vacuum evaporation [50]. In the study of Hu et al. (2015), no lengthy or energy-intensive procedures were required and therefore their work is most comparable to our results. Corn stover was pretreated with NaOH and afterwards was washed to remove inhibitors. With this material, SSF with 5% solids loading was performed using *B. coagulans* LA204 and Cellic^®^ CTec2 (30 FPU g^−1^ stover). After 60 h, this process resulted in 29.9 g L^−1^ LA with an optical purity of 93.7% and 60% yield (g LA/ g of total pretreated biomass used). The process exhibited 0.50 g L^−1^ h^−1^ average productivity and 4.5 g L^−1^ acetic acid were measured [51]. In our study, regarding the substrate RS-LX, a higher LA titer was achieved in less process time with an average productivity of 1.2 g L^−1^ h^−1^ in 32 h, and 0.83 g L^−1^ h^−1^ in 48 h. Furthermore, no acetic acid was measured and the produced LA had higher optical purity. The overall process yield of 56% (based on the theoretical quantity of monosaccharides present in the RS-LX substrate) was lower than that reported by Hu et al. (2015). Furthermore, a higher enzyme charge of 67 FPU g^−1^ dry matter was used. However, since twice as many solids (10 TS%) were employed in our process with RS-LX, the water input of the fermentation process was most likely reduced.

When considering the PSSF fermentation with DCS-LX, the lactic acid concentration of 18.3 g L^−1^ was considerably lower than the value obtained by Hu et al. (2015). However, the process took only half the amount of time with an average productivity of 1.02 g L^−1^ h^−1^ (12 h prehydrolysis followed by 18 h of fermentation). Also, our result was positively enhanced by the fact that biogas and LX-Lignin have already been successfully obtained from the substrate prior to its utilization for LA-fermentation. Thus, while the cascade use of this biomass has already generated two valuable products, 61% of the lactic acid titer reported by Hu et al. (2015) can still be obtained by performing PSSF, and 71% by performing SHF.

To further increase the LA titer, Hu et al. (2015) performed fedbatch SSF and produced 97.6 g L^−1^ LA in 60 h with an optical purity of 98.1%. The solid content was increased to 14.4% over the course of the fermentation [51]. By performing fedbatch SHF and employing *B. coagulans* P38, Peng et al. (2013) achieved 180 g L^−1^ L-LA in 75 h, which is, to our knowledge, the highest LA concentration from corn stover currently found in the literature [52]. However, even in this case, the corn stover pretreated with acid was concentrated in advance. Nevertheless, fedbatch fermentation is a promising process route to increase the LA titer while reducing the water-to-solid ratio. The SSF and PSSF results of the LX-Substrates presented in this report provide an excellent basis for planning future fedbatch experiments.

In conclusion, a new biorefinery concept was presented in this study which follows a value-adding cascade approach to gain several products from the renewable resources rye or energy corn. After harvest of the first and biogas generation from the latter, the lignocellulosic residues were further utilized for the novel LX-Pretreatment. While gaining chemically usable LX-Lignin, LX-Cellulose was also created which contains negligible inhibitors and an increased amount of glucan, making it an ideal candidate for fermentation studies. By employing thermophilic *B. coagulans* 14-300, LA of a high optical purity could be successfully obtained from LX-Cellulose of both substrates via simultaneous fermentation and saccharification. Results of the fermentation experiments with RS-LX from rye straw are promising and exceeded data from comparable research. To further increase the LA concentration, future studies will focus on a fedbatch SSF approach. Furthermore, scale-up experiments will be conducted to perform studies on the downstream of the fermentation broth to obtain chemically pure L-LA.

## Figures and Tables

**Table 1:** Lignocellulose composition of raw and LX-pretreated RS and DCS (FS = feedstock, *as* = acid soluble, *ais* = acid insoluble, LX-C = LX-Cellulose, LX-L = LX-Lignin) ^1^.

	RS			DCS		
	FS (%)	LX-C (%)	LX-L (%)	FS (%)	LX-C (%)	LX-L (%)
ash	5.3	4.3	0.5	8.6	7.9	1.4
cellulose	38.0	60.1	0.6	29.3	38.4	0.5
hemicellulose	25.5	7.7	3.4	22.4	8.5	2.4
lignin (*as*)	7.1	2.9	2.4	5.2	2.0	4.1
lignin (*ais*)	20.0	24.1	90.3	28.3	38.0	88.2
acetic acid	2.6	0.7	1.3	2.2	1.1	1.0
not identified	1.5	0.2	1.5	4.0	4.1	2.4

^1^ Analysis according to the laboratory analytical procedures stated by the NREL.
